# The Lunatic Asylums of Bengal

**Published:** 1899-11-18

**Authors:** 


### THE LUNATIC ASYLUMS OF BENGAL.

Out of the five asylums which Bengal possesses for its native lunatics two of the largest show a marked decrease in 1898 in the number of patients admitted. This diminution is supposed to be due to the fact that the population suffered far less from famine than in the previous year, consequently not only was there less mental aberration owing to want of food, but those lunatics usually maintained privately by relatives were not, as in 1897, driven into the asylums by the general distress prevailing. A satisfactory sign of the efficacy of the treatment given is afforded by the fact that at Dullunda, one of the largest asylums in Bengal, there were fewer readmissions, and the number of recoveries showed a marked increase. The admissions to hospitals and the daily average sick had decreased by 122, which is attributable to improvement in sanitation, to the issue of prophylactics, and to the maintenance of the "infirm gang," which facilitates the early recognition and care of those losing in health. The desirability of the maintenance of an "infirm gang" in lunatic asylums is a matter at present under discussion. It is objected to by some superintendents on the ground that it gives a false idea of the health of the asylum, and because all deviations from normal condition, whether fits, excitement, paralysis, or other ailments, should, it is maintained, be closely observed by the civil hospital assistant, and when necessary relegated to the hospital or cells. But after inspecting the two asylums—Dullunda and Dacca—where this system is at work, the Inspector-General of Civil Hospitals has come to the determination that it should be maintained in all the lunatic asylums at the discretion of the superintendents. The "infirm gang" simply means that weakly-looking persons are more frequently weighed, receive some extra diet, and are given tonics and drugs. By thus keeping the weakly and aged under closer observation serious disease is often warded off. It is therefore desirable as a part of general management in an institution in which all the inmates are really diseased; and at Berhampore the superintendent has been asked to introduce the system and report the result after six months. The management of the European lunatic asylum at Bhawanipur, where there is accommodation for 42 inmates, appears to have been perfectly satisfactory, and a lawn tennis ground has lately been added for the benefit of the patients.

